# Executive function and effortful control—Similar and different evidence from big data analysis

**DOI:** 10.3389/fpsyg.2022.1004403

**Published:** 2022-12-14

**Authors:** Soo Eun Chae

**Affiliations:** Department of Education, Art and Humanities College, Gangneung–Wonju National University, Gangneung-si, South Korea

**Keywords:** executive function (EF), effortful control (EC), big data analysis, bibliographic information, Delis–Kaplan Tests of Executive Functioning System, Adolescent Temperament Questionnaire

## Abstract

**Introduction:**

The current study explored commonalities and similarities between executive function (EF) and effortful control (EC).

**Methods:**

The major empirical studies published between 2013 and 2022 in the World of Science (WoS) was collected. The bibliographic information was systematically analyzed.

**Results and discussion:**

(1) EC is the efficiency of executive attention that incorporates inhibitory control (IC), attentional control, activation mainly related to temperament. On the other hand, EF is the efficiency of self-directed action that encompasses IC, working memory (WM), and shifting/cognitive flexibility in particular focuses on the cognitive aspect. (2) EF research has overwhelmingly outnumbered EC research (2,000 EF studies vs. 50 EC studies per year). (3) According to a co-word analysis with keyword co-occurrences, the subject of preschool students and individual differences co-occurred in EF studies. (4) EC usually occurs with working memory and early childhood. In the more detailed analysis of the articles, the EF and EC studies used younger subject groups than older subject groups. EC studies were especially likely to use subjects in early childhood. (5) The Delis–Kaplan Tests of Executive Functioning System (D-KEFS) was the most commonly used test for EF. In contrast, the EC used self-report surveys such as the Adolescent Temperament Questionnaire (ATQ). This research illustrates and discusses key findings in the EC and EF data and provides suggestions for future study directions.

## Introduction

Self-regulation (SR) has been an important topic in learning and education for the past 130 years since Hall (1891) mentioned a “volitional” reaction as a concept instead of an “unconscious” reaction (Post et al., 2006). SR has traditionally been described in the context of educational and settings, as the ability to comply with a request” (Kopp, 1982), that results in initiating and ceasing activities. More recently, such ideas were expanded and specified to focus on goal-directed activities (Inzlicht et al., 2021). Given the idea, SR can be defined as activities to achieve goals in the context of human learning and socialization. These activities aims to develope both tempermantal and cognitive aspects.

The main constructs of self-regulation are executive function (EF) and effortful control (EC). EF is a self-directed action necessary in selecting and creating a goal, and it refers to implementing the goal and maintaining the behavior toward the goal (Baggetta and Alexander, 2016). Researchers note that EF is a construct composed of the following main components: (1) inhibitory control (IC), (2) working memory (WM), and (3) shifting/cognitive flexibility (Baggetta and Alexander, 2016). On the other hand, EC is “the efficiency of executive attention, including the ability to inhibit a dominant response, to activate a subdominant response, to plan, and to detect errors”(Rothbart and Bates, 2006, p. 129). Therefore, EC includes underlying constructs of (1) IC, (2) attentional control, and (3) activation. EC pertains more to emotional activities in nature and is a concept particularly focused on temperament. Given the conceptual definition, inhibition is a common notion penetrating EF and EC. In addition to structural similarity, EF and EC share a functional similarity: executive attention (Zhou et al., 2012). Due to this conceptual and functional similarity, one can often see an overlap in the use of EF and EC measurement tools. For instance, Go/No Go and Stroop testing are representative tools commonly used in EF (e.g., Belghali et al., 2022) and EC (e.g., Lengua et al., 2007). However, despite the conceptual similarities, there are differences between EF and EC studies. EF is primarily associated with self-regulating activities governed by a cognitive-psychological approach, the so-called “cool system” (Mischel et al., 2003). On the other hand, researchers have studied EC with the “hot system,” i.e., more emotion-laden regulatory activities. One core construct missing in EF research but not EC research drives this difference: working memory (Zhou et al., 2012). For instance, working memory is the most crucial cerebral activity in reasoning and academic performance (Gilhooly, 2004) and is relevant to attention (Gioia et al., 2002).

More recently, Gagne (2017) used temperament-based and neural systems approaches to distinguish between EC and EF. We can easily understand EC from a temperament-based approach, whereas EF needs a more neural systems approach. When understanding those concepts from self-control perspectives, the EF IC underlies cognitive functions, but the EC IC underlies emotional temperament dimensions (Liew, 2012). Regardless of academic history and trends, educational practices in the field use both concepts interchangeably (Gagne, 2017). Some scholars even argued for synthesizing both perspectives (Liew, 2012).

As described above, the distinction between EC and EF seems complicated due to the difficulty distinguishing between cognitive–emotional development and the commonality of measures and instruments. Existing literature does not address these problems sufficiently from a systematic data-based review. Thus, the current study explores these problems from several points. First, we review EF and EC studies to understand people circumvented by drastic technological, social, and pathological changes over the past ten years (2012–2022), such as those confronting online blended learning. Advances in research have led to the development and introduction of new psychometric measurements. In addition, a systematic analysis of the relevant literature is necessary to figure out more scientifically the commonalities and/or similarities between EF and EC. The current study drives these research gaps with the following specific research questions.

In the general educational context and for typically developing human beings, what are the similarities and differences between EF and EC regarding:

1. The number of publications by year?
2. Study characteristics revealed in the keywords?
3. Definitions?
4. Instruments and subjects?

Therefore, this study clarifies the conceptual and psychometric differences between EF and EC through big data-based analysis. However, this effort does not argue against a conceptual distinction between EF and EC. Instead, the present study reveals how to explain EF and EC under the umbrella term of self-regulation. Furthermore, this clarification could function as a base to suggest how to synthesize these two concepts in the field.

## Methods

### Search process

I used several search parameters and steps to drive an adequate dataset for answering the research questions. First, I collected studies from the Web of Science (WoS) database with the following search parameters: published since 2013 in peer-reviewed academic journals stamped with Social Science Citation Index (SSCI), or Science Citation Index (SCI), or Art and Humanity Citation Index (A&HCI) because the indices already screen quality studies. I only used English, human learning and performance, empirical studies in nature, and behavioral or neuroimaging instruments as search terms to represent the research topics. For instance, I excluded studies using meta-analysis on the effects of EF and EC (Sung et al., 2022) to avoid redundancy in the meta-analysis and empirical studies. Second, because this review’s principal goal was to find commonality and distinction between EF and EC in their concepts and operations, I created two data pools in the keywords: one containing executive function and another containing effortful control. The initial search process resulted in a collection of 17,038 EF studies and 482 EC studies. I downloaded the data on May 4, 2022.

**FIGURE 1:**
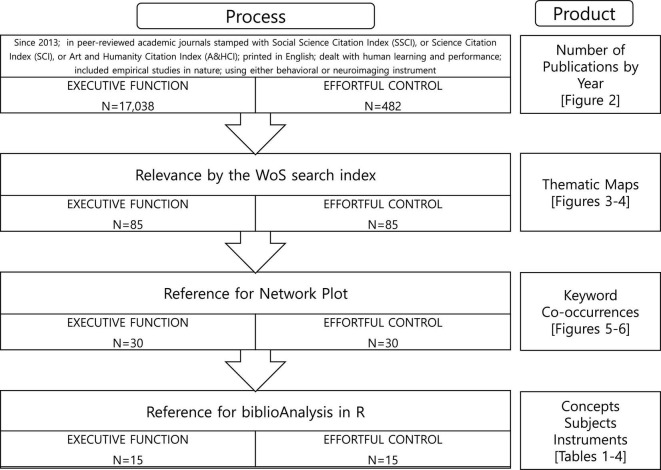
Data filtering process and the products using the data pool for executive function and effortful control studies.

Due to the many retrieved articles, the next step was to narrow the initial data pools to manageable levels. Thus, I filtered the EF studies based on their inclusion in the “HIGH H INDEX” category offered by the WoS database. This second step resulted in 85 EF articles for generating thematic maps. Finally, I further narrowed the datasets for more analyses (keyword co-occurrences, concepts, subjects, and instruments). Figure 1 summarizes the data filtering steps.

### Analysis

I obtained the number of publications by year from the initial search data from the WoS, which included 17,038 EF and 482 EC studies. To figure out study characteristics in the keywords, I considered 85 articles with high ranks according to the WoS search index for the EF and EC pools, respectively. First, I analyzed these pools’ keywords and obtained thematic maps. Next, I extracted keyword co-occurrences for the EF and EC pools with 30 top high-ranked articles. Finally, I used the Bibliometrix package in R (Aria and Cuccurullo, 2017) to map the themes and co-occurrences with keywords from the pools.

In addition, to address differences in the concepts/operations, subjects, and instruments, I analyzed 15 highly-referenced articles from each of the EF and EC collections. Then, I extracted conceptual similarities and differences by reviewing the collected papers. Finally, after the physical screening, I examined the EF and EC measurements and population groups.

## Results and discussion

### Annual publication

**FIGURE 2:**
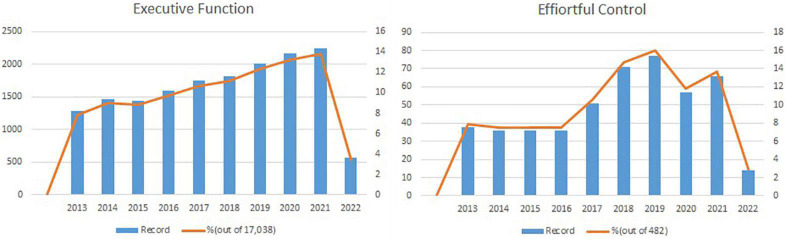
Amount of publications for executive function studies **(left)** versus effortful control studies **(right)** by year (2013–2022).

Figure 2 illustrates the initial search process, where bar charts represent the number of publications by year, and the line charts are the percentage of publications within that year out of the total published articles over the recent decade. As displayed in the left chart, the number of EF study publications steadily increased from approximately 1,400 to 2,200. On the other hand, the annual EC publications remained similar from 2013 to 2016 (about 40), then almost doubled from about 40 in 2016 to 80 in 2019. The increment was again steady afterward. Regardless of the trend in the annual publication rates, the number of total publications over the decade contrasts between EF and EC. While EC studies are about 50 per year, EF studies are approximately 2,000 per year, i.e., 400 times more than EC studies.

### Study characteristics revealed in keywords

#### Thematic maps using keywords

**FIGURE 3:**
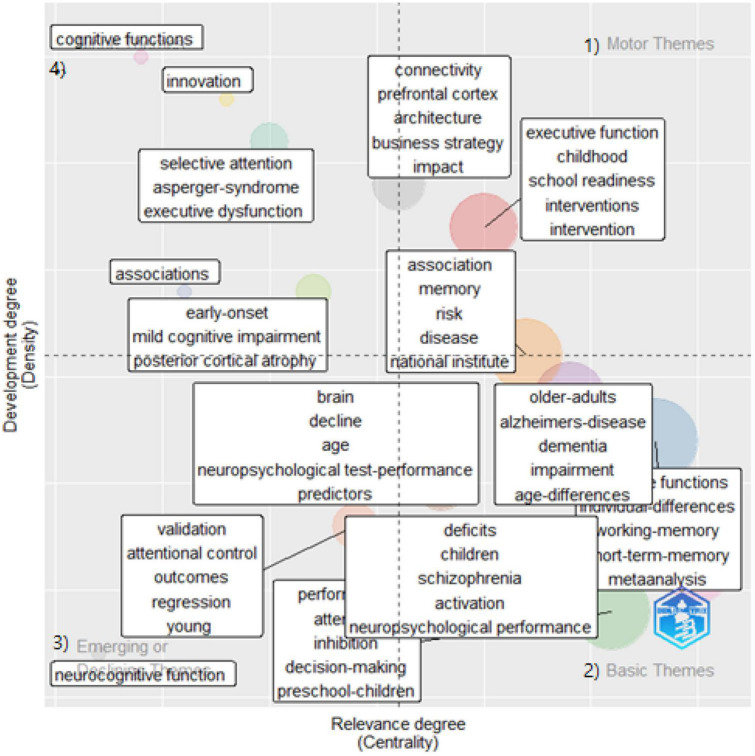
Thematic map of 85 executive function studies (2013–2022). Each quadrant refers to (1) a motor theme, (2) a basic theme, (3) an emerging/declining theme, and (4) a specialized/niche theme.

**FIGURE 4:**
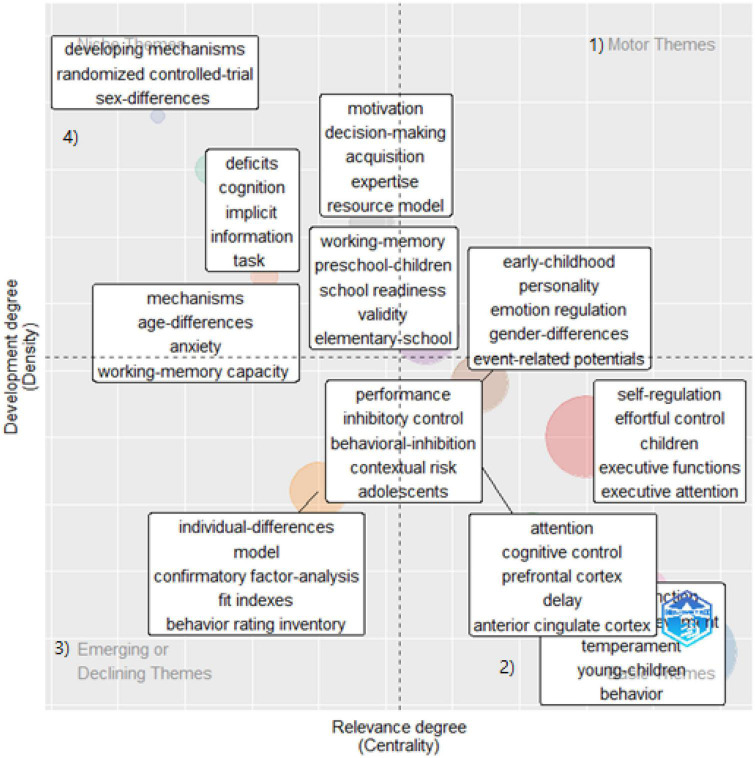
Thematic map on 85 effortful control studies (2013–2022). Each quadrant refers to (1) a motor theme, (2) a basic theme, (3) an emerging/declining theme, and (4) a specialized/niche theme.

I mapped clusters of keywords on a two-dimensional diagram covering density and centrality to enable an understanding of significant research trends (Figures 3, 4). Centrality refers to the degree of interaction a cluster has with other parts of the network. Density means the degree to which a particular keyword appears in the content several times (Hu et al., 2013). The thematic map is an intuitive plot that locates the themes according to the quadrant: (1) the upper right quadrant refers to the motor theme, (2) the lower right presents the basic theme, (3) the lower left quadrant means emerging and declining themes, and (4) the top left quadrant is the specialized/niche theme.

Figure 3 shows the thematic map for the discourse in executive function studies. The motor themes of the EF studies (quadrant 1) conveyed school-readiness interventions for children. In addition, I observed a prevalence of basic (quadrant 2) and niche themes (quadrant 4). The basic themes covered three chunks: the first chunk regards older adults’ cognitive impairment (e.g., Alzheimer’s, dementia), the second chunk pertains to children’s deficits (e.g., neuropsychological performance and schizophrenia), and the third chunk concerns memory (short-term and long-term). Overall, the basic themes retrieved from the EF studies were relevant to age-specific cognitive malfunctioning. Niche themes (quadrant 4) were pertinent to selective attention, Asperger syndrome, and executive dysfunction.

Figure 4 shows a thematic map highlighting the discourse in effortful control studies. The hot topics of the EC studies, presented in motor themes (quadrant 1), conveyed personality and emotional regulation in early childhood. The “hot” system weighing temperament and emotion seemed closely related to the EC studies, as noted by Mischel et al. (2003). As opposed to older adults as focal research subjects in EF studies, the basic themes for EC studies (quadrant 2) comprised three clusters mainly dealing with young children. The first keyword cluster was young children’s temperament, the second cluster regarded children’s EF and attention, and the third covered petrophysical functioning (e.g., prefrontal cortex and anterior cingulate cortex) concerning attention and delay. Developing mechanisms, randomized control, and cognitive deficits were niche themes (quadrant 4) in the EC studies, i.e., themes for specific fields.

In addition, I located three chunks of themes in the center of the chart regarding the relevance degree of EF studies. The first chunk pertained to expertise and decision-making. The second included school readiness for preschool and elementary school students. The last chunk was about adolescents’ inhibitory control and performance, which showed sparse density compared to the first two chunks. Finally, confirmatory factor analysis for the EC behavior rating inventory resulted in emerging or declining themes (quadrant 3).

#### Co-word analysis with keyword co-occurrences

**FIGURE 5:**
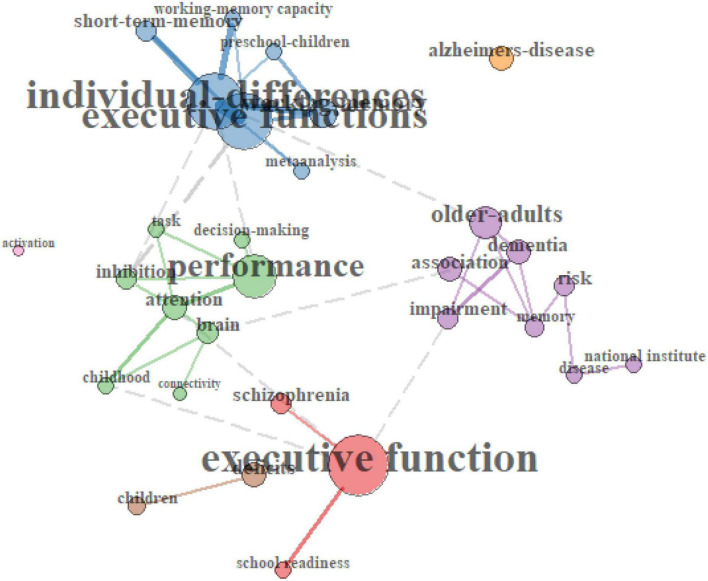
Keyword co-occurrence plots on 30 most cited executive function studies (2013–2022).

A program generated a visual word map of co-word networks to uncover links between concepts through term co-occurrences. As one can observe from Figure 5, four major chunks of keywords emerged from the 30 most cited EF studies according to the degree to which the keywords were likely to occur together. Individual differences in preschool children appeared, and performance co-occurred with inhibition, brain, and attention in childhood. Schizophrenia and school readiness also strongly co-occurred with executive function. Finally, the older adult presented together with dementia and memory impairment.

**FIGURE 6:**
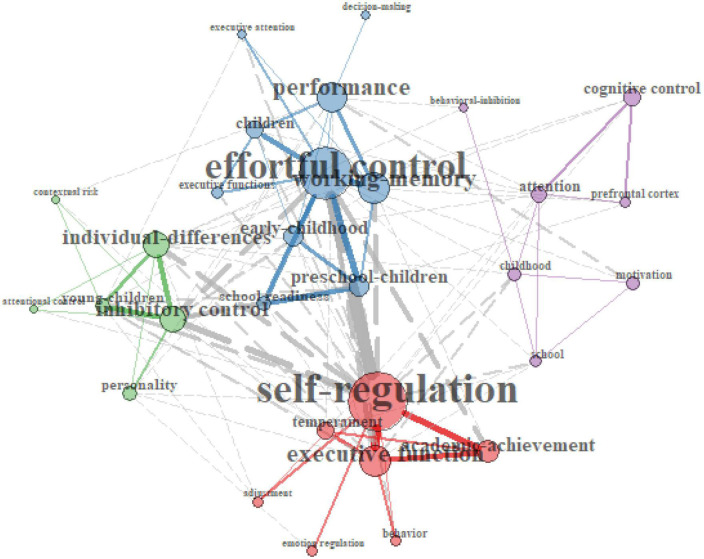
Keyword co-occurrence plots on 30 most cited effortful control studies (2013–2022).

Likewise, Figure 6 shows three co-occurring chunks of keywords. Effortful control arose with working memory, early childhood, and preschool children. Self-regulation also comprised a big keyword chunk with achievement and temperament in this study pool. Finally, individual differences, IC, and personality co-occurred and were strongly related to EF. These trends were similar to what I found in the thematic maps (Figures 3, 4).

### Concepts of executive function and effortful control

In addition to the above structural analysis for recent EF and EC studies, I performed a semantic analysis to comprehend academic definitions of these two constructs. I retrieved 15 top-cited articles from each study pool. The explicit descriptions in the articles are as follows (Tables 1, 2). According to these references, the most common use of adjectives defining EF included “goal-directed” (e.g., Benson et al., 2013), “domain-general” (e.g., Lucas et al., 2013), and “task-related” (Gijselaers et al., 2017). The components or processes for defining EF were “self-regulation,” “control,” “working memory,” “inhibition,” “planning,” “attention,” and “shifting” (e.g., Rhodes et al., 2016). EF is a multifaceted construct comprising higher-order and lower-order functions. For instance, Gijselaers et al. (2017) viewed EF as a hierarchical construct of common EF and EF-specific variation. In addition, “cognitive” processes (e.g., Niermeyer et al., 2019) were salient for attributes. This overall trend is consistent with Zhou, Chen, and Main’s study Zhou et al. (2012). However, other studies also mentioned “emotional” and “social” processes (e.g., Lima et al., 2014). The most cited articles defined EF as a cognitive process underlying goal-directed and task-related behavior and a multifaceted construct, including self-regulation, working memory, inhibition, planning, attention, and shifting. The EF can also encompass emotional and social regulatory processes.

**TABLE 1:** Explicit definitions of the executive function retrieved from 15 most cited articles.

No.	First author	Year	Definition
1	Benson	2013	• The processes that underlie *goal-directed behavior* including self-regulation, planning, working memory, response inhibition, and resistance to interference (Carlson et al., 2013)
2	Lucas	2013	• *Domain general skills* that enable the planning and control of their behavior • These skills involve cognitive flexibility, inhibitory control (IC), and working memory
3	Semrud-Clikeman	2014	• A heterogeneous term frequently incorporates working memory, cognitive flexibility, planning, and organization (Nigg et al., 2002) • These skills refer to how a person understands situations rather than what the person knows
4	Rhodes	2016	• A broad term used to describe essential organizational processes that go beyond working memory to include a range of other strategic processes: Anticipation and deployment of *attention*, *impulse control and self-regulation, initiation of activity, working memory, mental flexibility, and utilization of feedback, planning ability, and organization, and selection of efficient problem-solving strategies* (Anderson, 2008)
5	Rhodes	2014	• A compendium of constructs comprising three core, dissociable components: *inhibition, working memory, and set-shifting* (Miyake et al., 2000; Lehto et al., 2003; Diamond, 2013), and several higher-level functions such as planning and problem solving (Diamond, 2013)
6	Niermeyer	2019	• A complex, multifaceted construct that consists of a set of higher-order cognitive abilities that allow an individual to engage in successful *goal-directed behavior* that is adaptive and socially informed (Stuss et al., 2001; Cummings and Miller, 2007; Lezak et al., 2012; Suchy, 2015)
7	Lundervold	2019	• *General-purpose control mechanisms* (Miyake et al., 2000) that serve to regulate cognitive processing, especially in complex and/or novel settings
8	Boschiloo	2014	• The functions necessary for *goal-directed behavior* (e.g., Best and Miller, 2010) • The literature describes a wide range of executive functions, such as inhibition, updating working memory, shifting, planning, organization skills, attentional control, and self-control (Alvarez and Emory, 2006; Best and Miller, 2010; Hofmann et al., 2012)
9	Martin-Perpina	2019	• The capacities for *formulating goals, planning, and carrying out plans* effectively; essential for independent, creative, and socially constructive behavior
10	Lima	2014	• A set of cognitive skills that enable the individual performance of voluntary *actions to orient goals*, encompassing control processes in cognitive, emotional, and social areas
11	Gijselaers	2017	• *Common EF* is the ability to *manage the tasks at hand and the task-related information* and use this information to guide and steer lower-level processing • EF-specific variation is the variation that remains after controlling for common EF variation • When controlling for common EF variation, there is only a specific variation for updating and shifting (Miyake and Friedman, 2012) • This finding means that the common EF ability is a basic need for all three EFs and is especially important for inhibition, as no EF-specific variation remains after controlling for common EF (Miyake and Friedman, 2012)
12	Rosas	2017	• These are psychological processes involved in the *conscious control of thought and action* (Zelazo and Müller, 2011). • This group is a family of functions we use when we need to concentrate, and following our initial impulses is inappropriate (Diamond, 2012) • The main components of EF are IC, working memory (WM), and cognitive flexibility (CF) (Diamond, 2013)
13	Ljubin Golub	2016	• A set of correlated but separable control processes that *regulate* lower-level cognitive processes in support of *goal-directed* behavior (Friedman et al., 2008): inhibition of automatic or prepotent response and updating working memory representations, and shifting/switching between tasks or mental sets (Friedman et al., 2008) • It also includes sustained and selective attention (Alvarez and Emory, 2006), and dual-tasking (Logie et al., 2004)
14	Kavanaugh	2016	• A collection of “top-down” *control* and self-regulatory processes required to *obtain goals and objectives* (Barkley, 2012; Diamond, 2013)
15	Taha	2017	• An umbrella term for the *management, regulation, and control* of cognitive processing (Lezak, 2004, p. 611)

**TABLE 2:** Explicit definitions of effortful control retrieved from 15 most cited articles.

No.	First author	Year	Definition
1	Kim	2013	• The capacity to *suppress* deliberately and voluntarily *a dominant or prepotent response and perform a subdominant response* is a key aspect of children’s *temperament* (Derryberry and Rothbart, 1997; Rothbart and Bates, 2006) and personality (Caspi and Shiner, 2006)
2	Duckworth	2013	• The ability to *inhibit a dominant response to perform a subdominant response* (Rothbart and Bates, 1998, p. 137)
3	Lipsey	2017	• Involves volitional behavioral regulation related to aspects of *temperament* (Kochanska et al., 2000); *suppression of impulsive or premature responses* when required by a task
4	Bao	2015	• The efficiency of executive attention, including the ability to *inhibit a dominant response and/or activate a subdominant response* and plan and detect errors (Rothbart and Bates, 2006, p. 129)
5	Studer-Luethi	2016	• A *temperament* factor in childhood represents the developmental process underlying conscientiousness, naming it effortful control (cf. Ahadi and Rothbart, 1994; Blair and Razza, 2007) • Together, neuroticism and effortful control represent the two temperament categories: reactivity and self-regulation (Rothbart et al., 1994)
6	Wang	2018	• A group of abilities concerning how well an individual could *inhibit a dominant response, activate a subordinate response*, plan, and detect errors (Rothbart and Bates, 2006)
7	Zeytinoglu	2017	• The regulatory component of *temperament* involves attentional processes that enable individuals to voluntarily *shift and focus their attention and inhibit or activate their responses* (Evans and Rothbart, 2007)
8	Di Norcia	2015	• *Delaying, slowing down motor activity, suppressing or initiating an activity* when required, lowering voice, and effortful attention
9	Lin	2019	• The ability to *inhibit a dominant* (motor, vocal, emotional, or cognitive) response and *activate a subdominant response* (Rothbart et al., 2003; Rueda, 2012): IC, effortful attention, conflict resolution, and the ability to identify and correct errors and plan actions (Kochanska et al., 2000)
10	Lin	2013	• A set of regulatory processes to *inhibit dominant* (but inappropriate) *responses*, perform subdominant (but avoidant) behaviors and *control attention* (Evans and Rothbart, 2007)
11	Sulik	2015	• The self-regulatory aspect of *temperament that supports volitional control* of attention, emotion, and behavior
12	Tiego	2020	• The efficiency of executive attention includes the ability to *inhibit a dominant response and/or activate a subdominant response* and plan and detect errors (Rothbart and Bates, 2006, p. 129)
13	Omura	2015	• The ability to *inhibit a dominant respons*e *to perform a subdominant response* and/or facilitate efficient executive attention: attentional, inhibitory, and activation control (Rothbart et al., 2000, 2001)
14	Zorza	2013	• A basic dimension of *temperament* that mediates between voluntary control of behavior and regulation of emotional reactivity (Derryberry and Rothbart, 1997)
15	Cerda	2014	• Involves the abilities to enjoy activities of *minimal intensity, to shift and focus attention deliberately, and inhibit or initiate a response* as required by particular circumstances (Putnam et al., 2006; Gartstein et al., 2012)

While EF regarded more “what to do,” EC highlighted “what not to do.” The most cited articles often mentioned “inhibit a dominant response,” “suppress impulsive or premature responses,” and “self-regulation” in their definition. In addition to these highlights on IC over premature and unnecessary responses, studies included “activation of a subdominant response” and “reactivity” as core components of EC. Following Zhou et al.’s (2012)’ study, definitions and operations indicated that EF and EC’s commonality often included inhibition as a core construct. In addition, researchers discriminated EC from EF because EC is more of a “temperament” (Lipsey et al., 2017). I also found this trend in the current analysis.

### Instruments and subjects

The common test for EF is the Delis–Kaplan Tests of Executive Functioning System (D-KEFS), which includes Wisconsin Card Sorting (to measure shifting), Trail Making (to measure IC), and the verbal fluency test (to measure working memory) (see Table 3). Otherwise, researchers used similar tasks to measure the underlying constructs of shifting, inhibitory control, and working memory. For instance, Benson et al. (2013) examined children’s shifting ability with the “Bear/Dragon” game, similar to the “Simon Says” game. Other studies often measured shifting ability with a card sorting test (e.g., Lucas et al., 2013).

**TABLE 3:** Instruments and subject of executive function retrieved from 15 most cited articles (Supplementary Appendix).

No.	First author	Year	Subject	Age or grade	*N*	Instrument
1	Benson	2013	Child	3.5 years	24	• Response Conflict-Executive Functioning scale = Bear/Dragon + Grass/Snow + Dimensional Change Card Sort
2	Lucas	2013	Child	Preschool	144	• Dimensional Change Card Sort (set-shifting) • Day/Night (IC) • Eight Boxes (working memory)
3	Semrud-Clikeman	2014	Child	8.5–17.5 years	108 = 38 Control + 36 Autism + 31 Non-verbal learning disabilities	• Delis-Kaplan Tests of Executive Functioning System *(D-KEFS*) (Delis et al., 2001) = Card Sorting + Trail making + Verbal Fluency
4	Rhodes	2016	Adolescent	12–13 years	63	• Cambridge Neuropsychological Test Automated Battery (CANTAB) (Morris et al., 1987) = SWM (Spatial Working Memory) + Stockings of Cambridge (planning) + Stop-Signal (inhibition) + ID/ED (attention set-shifting).
5	Rhodes	2014	Adolescent	12–13 years	56	• Spatial Working Memory (SWM; working memory) + Stockings of Cambridge (SOC; planning) + Stop-Signal (inhibition) + ID/ED (attention set-shifting)
6	Niermeyer	2019	Older Adult	69.19 years	110	• Delis–Kaplan Executive Functioning System battery (*D-KEFS*; Delis et al., 2001)
7	Lundervold	2019	Adult	30 years	63 ADHD + 73 Control	• PASAT (Working Memory), Color-Word Interference Test (Response Inhibition)
8	Boschiloo	2014	Adolescent	12–18 years	173	• Objective: Sorting Test and the Tower Test from the Delis-Kaplan Executive Functioning System (*D-KEFS*) (Delis et al., 2001) • Subjective: Behavior Rating Inventory of Executive Function—Self Report Version (BRIEF-SR) (Guy et al., 2004)
9	Martin-Perpina	2019	Adolescent	11–18 years	977	• Dysexecutive Questionnaire (DEX-SP) (Wilson et al., 1996)
10	Lima	2014	Child, Adolescent	6–16 years	31 Epilepsy + 35 Controls	• Wisconsin Card Sorting Test (*WCST*)
11	Gijselaers	2017	College student	18–80 years	4,945	• Trail Making Test (TMT; Army Individual Test Battery, 1944) • Substitution Test (ST) (symbol digit modalities test by Smith, 1991) • N-back task (NBT; Lezak et al., 2004)
12	Rosas	2017	Child	5.5 years	109	• Hearts & flowers (General EF measures) • Stroop animal (Cognitive inhibition) • Bzz! (Behavioral inhibition) • Torpo (Visual working memory) • Geometric figures (Cognitive flexibility)
13	Ljubin Golub	2016	College student	20 years	87	• Verbal fluency task • Stroop task
14	Kavanaugh	2016	Child	6–12 years	76 No-Neuropsychology + 75 Neuropsychology	• COWAT-FAS • Trail Making Test-B • Stroop Color • Word Test-Children’s Version • Wisconsin Card-Sorting Test • Rey Complex Figure Test-Copy Condition
15	Taha	2017	Child/w asthma	12.46 years	27 Asthmatic + 30 Normal	• Wisconsin Card Sorting Test (*WCST*)

When it comes to EC, the major research instrument is the self-report survey. For instance, six out of 15 EC studies used the Adolescent Temperament Questionnaire (ATQ) (e.g., Lin et al., 2013; Zeytinoglu et al., 2017) or the Early Adolescent Temperament Questionnaire (EATQ). Evans and Rothbart (2007) developed the original ATQ in 35 items capturing (1) attention control (12 items), (2) activation control (12 items), and (3) IC (11 items). Each item asks the respondent to indicate their agreement with a statement (e.g., “Although the assignment is hard, I can finish it on time”). Later, researchers revised and published a shorter version with 17 items for adolescents. The next instrument researchers frequently used was the Delay-of-Gratification, applied in three studies (e.g., Duckworth et al., 2013; Kim et al., 2013; Lin et al., 2019).

In terms of subject groups, EC studies (Table 4) involved very young subjects such as infants (Kim et al., 2013) or toddlers (Sulik et al., 2015; Lin et al., 2019). In contrast, EF studies (seven out of 15) used children (Benson et al., 2013) as a subject group. This phenomenon seems to pertain to the cognitive development process of humans. In childhood, corresponding to the early stage of development, the brain is less myelinated and thus shows very distracted brain activity (Brydges et al., 2013). As a result, children’s IC for minimizing and simplifying unnecessary tasks to achieve goals is weaker than adolescents’ (Atherton et al., 2020). In addition, effortful control develops around two years of age and rapidly in infancy (Kim et al., 2013).

**TABLE 4:** Instruments and subject of effortful control retrieved from 15 most cited articles (Supplementary Appendix).

No.	First Author	Year	Subject	Age or grade	N	Instruments	
1	Kim	2013	Infant in a two-parent family	①T1 38 month ②T2 52 month	100	• ①Assessments of EC “Hot” Function: Delay-of-*Gratification* Tasks	• ②EC “Cool” Functions: Motor Inhibition, Go-No Go, Effortful Attention Tasks
2	Duckworth	2013	①Youth ②Early child	①5th grade ②4 year	56	• ①Reward-related impulses/CBQ attention focusing	• ②Delay of *gratification*
3	Lipsey	2017	Early child	pre-K	608	• Whisper and Turtle-Rabbit tasks	• Teacher Ratings of Cognitive Self-Regulation
4	Bao	2015	Adolescent	7th–9th grade M = 13.53 year	2,758	• Adolescent Temperament Questionnaire-Revised (*ATQ*-R, Ellis and Rothbart, 2001)	
5	Studer-Luethi	2016	Child	2nd grade M = 8year. 3 month	99	• Child’s Working Memory (WM) task	• Teachers’ ratings (EC) • Parents’ ratings (EC, neuroticism)
6	Wang	2018	Adolescent	6th–8th grade	850	• Early Adolescent Temperament Questionnaire-Revised (*EATQ*-R, Capaldi and Rothbart, 1992)	
7	Zeytinoglu	2017	Mother	19–58 year	278	• Adult Temperament Questionnaire Short Form (*ATQ*; Evans and Rothbart, 2007)	
8	Di Norcia	2015	Early child	25–41 month	74	• Reverse categorization • Musical box • Slowing down • Motor activity • Lowering voice • Clean-up	
9	Lin	2019	Early child	4–6 year	244	• EC(Hot): Snack *Delay task*,Toy Delay task (Kochanska et al., 2000)	• EF(Cool): Stroop, K-CPT
10	Lin	2013	Undergraduate (adolescent)	19.45 year	320	• Adolescent Temperament Questionnaire (*ATQ*) (Evans and Rothbart, 2007) = activation control (12 items) + attention control (12 items) + IC (11 items)	
11	Sulik	2015	Early child	4.49 year	106	• Bird and Dragon • Knock-Tap • Gift Wrap • Continuous Performance Task	
12	Tiego	2020	Early adolescent	11 year	136	• Early Adolescent Temperament Questionnaire-Revised (*EATQ*-R) = self-report + parent-report	
13	Omura	2015	Adult	20.42 year	27	• AX-CPT during EEG (similar to the Go/No Go task)	
14	Zorza	2013	Adolescent	12–14 year	359	• Early Adolescence Temperament Questionnaire–Revised Self Report (*EATQ*-R self-report; Ellis and Rothbart, 2001)	
15	Cerda	2014	Child	1st grade	744	• Walk-a-Line • Star Telephone Poles • Circle • (IC, task accuracy)	

In addition, there is a shared belief in establishing EC early as possible for satisfactory human socialization and schooling (Eisenberg et al., 2003). For instance, psychologists have chosen infant EC as their research topic following the EC’s critical period and its ripple effect on infants’ lives (e.g., Duckworth et al., 2013; Kim et al., 2013; Lipsey et al., 2017). In contrast to the research gap between EC and EF in using infants as study subjects, researchers used adolescents with a similar frequency (5 out of 15) between EF (e.g., Rhodes et al., 2016) and EC studies (e.g., Bao et al., 2015). Researchers were less likely to use adult subject groups for EC and EF studies; however, I found one more article in the EF study pool than in the EC study pool. In sum, the EF and EC studies used younger subjects more often than older subjects. In addition, EC studies were especially likely to use subjects in early childhood.

## Key findings

This study explored the common attributes and differences between EF and EC based on the results of major empirical studies published between 2013 and 2022. As a result of big data analysis using bibliographic information published in the World of Science (WoS), major published papers found a slight difference between EC and EF in terms of concepts, measures, instruments, and subjects of use.

### Hot effortful control and cool executive function

As per the definition, the efficiency of executive attention that incorporates inhibitory control (IC), attentional control, activation mainly related to temperament. On the other hand, most EF studies focused on the cognitive rather than the affective aspect. The keyword analysis also showed a slightly more pronounced difference between the two research streams. According to the keyword thematic topic analysis, in the EF studies, cognitive keywords such as “working memory” and “short-term memory” appeared as base themes. On the other hand, the EC studies include temperament as the base theme leading the basic flow of the study.

Metcalfe and Mischel’s (1999) hot versus cool framework explains the given conceptual differences well through a hot/cool system; humans have a two-fold interactive processing system. The hot system is the “go” system because it follows an emotional process and responds immediately and simply. It decreases under stress and is necessary for the control of external stimuli. On the other hand, the cool system follows a cognitive process, develops slowly and late, and has the nickname “know” system. When stressed, the cool system becomes a stimulus rather than an activation and is necessary for voluntary control. The EF functions based on a cool system, whereas the EC is based on a hot system.

Regarding measures and instruments, the EF–EC distinction needs further discussion. Indeed, the present analysis of the measures showed overlaps between the two concepts. For example, major EF studies used such comprehensive batteries as D-KEFS, which highly rely on cognitive interaction activity time, such as the Sorting Test and Tower Test. At the same time, there was considerable use of performance tests (e.g., Go/No Go, Trail-Making) that measure immediate response in EF studies. EC researchers also switched between instruments based on hot and cool systems. For instance, Kim et al. (2013) used a representative hot system-based measure called “Delay of Gratification” to measure EC and a cool system-based measure such as “Go/No Go.” The Adolescent Temperament Questionnaire (ATQ) (Evans and Rothbart, 2007), which frequently appears in EC research, is based on effortful control, consisting of three sub-constructs: activation control, attention control, and IC. Attention control is close to cerebral activity, and IC is an item measuring temperamental activity. It is challenging to differentiate between these two constructs due to the ambiguity of the hot–cool systems in the EC and EF measurement tools and their use. Nevertheless, we can understand this commonality in the same context as what was argued by the existent literature (e.g., Liew, 2012; Gagne, 2017).

### Younger subjects used in effortful control studies than in executive function studies

A more noticeable difference was captured between the EC and EF studies concerning the study subjects. Statistically, participants’ ages in EC studies were lower than in EF studies. Researchers argue that EC of self-regulation abilities critically develops at 22–33 months of age (Bernier et al., 2010); some even claim 12–18 months as a critical period in EC development (Kochanska and Knaack, 2003). Thus, there seems to be an age difference between EC and EF development. Moreover, EC researchers predominantly used infants or toddlers in their studies. In contrast, EF studies used children older than the EC’s major study participants but still young. The EF and EC studies with this interest in children support existing studies (Montroy et al., 2016) that early stages of human development result in differentiated self-regulation.

There is a link between the age difference of study subjects and the main topics covered in EF and EC studies. For example, research topics that form a significant trend regarding EF were school readiness and interventions related to school adjustment. This finding is of interest to researchers considering that the subjects of EF studies are mainly children. In addition, the main keywords such as “emotion regulation,” “personality,” and “event-related” confirm the flow of EC research. One can infer emotion regulation and personality to accompany EC studies, considering the operational definition of EC frequently includes temperament. However, more direct measures such as the event-related instrument would be useful when researchers pay attention to babies before language development because the subjects’ self-report is unavailable, and their behaviors are not easy to interpret.

## Future directions

In this study, I explored the similarities and differences between EC and EF through big data analysis of major studies over the past decade. Still, undoubtedly, we need more work. Therefore, I derived several important future research topics in summarizing this study’s key findings.

In terms of publication numbers over the past decade, EF research has overwhelmingly outnumbered EC research (2,000 EF studies vs. 50 EC studies per year). Few researchers are studying self-regulation or IC from an emotional perspective, as few invoke EC. Most come from the EF perspective. The difference in publication number relates to the analysis results in which the academic and operational definition of EF often already includes the academic and operational definition of EC. Researchers know less about self-regulation in the hot system (EC). Furthermore, researchers have usually paid attention to EC as a way to solve emotional problems such as violence and delinquency in *children and adolescents* (Eisenberg et al., 2003). However, we must advance studies on the EC development of *older subjects* such as adults and the elderly.

It seems necessary to make EC and EF typography a broad spectrum. In other words, when and how we differentiate the EC and EF sub-constructs, it is essential to map them according to the stage of human development. One can start the discussion with the example of studies on inhibition, a key and basic construct of EF and EC. IC appeared to show individual differences around the age of one to two at the onset of toddlerhood (e.g., Montroy et al., 2016). If so, when will the remaining sub-constructs (working memory, shifting, planning, organization, and attentional control) become noticeably differentiated? The answer to this question will provide the basic idea needed to grow and develop EF and EC, a psychological construct that directly impacts academic performance. The answer depends on devising a program for children’s cognitive development or providing an educational environment.

Furthermore, it is necessary to broaden the understanding of determinants and outcome variables related to the development of EC and EF. For instance, one can ask how a person’s EC and EF develop or change before and after school age. How can EC and EF change when the person is situated in public education or home-schooling becuase these two environments involve different levels of temperament and cognitive engagement. This elaboration of the research questions may expand the existing EF and EC studies.

In addition, research on constructs of the agents also seems to need specification. For instance, the IC appeared to be a common core construct across EF and EC. At the same time, research has shown that the IC develops drastically during childhood. Thus, the systematic analysis of the IC studies targeting childhood would elaborate on the EF and EC differences and commonalities.

## Data availability statement

The raw data supporting the conclusions of this article will be made available by the authors, without undue reservation.

## Author contributions

The author confirms being the sole contributor of this work and has approved it for publication.
