# Supplementary material for: Executive function and effortful control—Similar and different evidence from big data analysis
Source: Front Psychol. 2022 Dec 14;13:1004403. doi: 10.3389/fpsyg.2022.1004403 (PMC9794866; doi:10.3389/fpsyg.2022.1004403)
Supplement: Supplementary file 1 [file Data_Sheet_1.docx]

**Appendix**

**The 15 Most Cited Executive Function (EF) Articles (2013–2022)**

Benson, J. E., Sabbagh, M. A., Carlson, S. M., & Zelazo, P. D. (2013). Individual differences in executive functioning predict preschoolers’ improvement from theory-of-mind training. *Developmental Psychology, 49*(9), 1615. [https://doi.org/10.1037/a0031056](https://psycnet.apa.org/doi/10.1037/a0031056)

Boschloo, A., Krabbendam1, L., Aben, A., de Groot, R., & Jolles, J. (2014). Sorting Test, Tower Test, and BRIEF-SR do not predict school performance of healthy adolescents in pre-university education, *Frontiers in Psychology (2014),* <https://doi.org/10.3389/fpsyg.2014.00287>

Gijselaers, H. J. M., Meijs, C., Neroni, J., Kirschner, P. A., & de Groot, R. H. (2017). Updating and not shifting predicts learning performance in young and middle-aged adults. *Mind, Brain, and Education, 11*(4), 190-200. <https://doi.org/10.1111/mbe.12147>

Golub, T. L., Rijavec, M., & Olcar, D. (2016). The relationship between executive functions and flow in learning. *Studia Psychologica, 58*(1), 47. <https://doi.org/10.21909/sp.2016.01.706>

Hu, C. P., Hu, J. M., Deng, S. L., & Liu, Y. (2013). A co-word analysis of library and information science in China. *Scientometrics, 97*(2), 369–382. <https://doi.org/10.1007/s11192-013-1076-7>

Kavanaugh, B. C., Gaudet, C. E., Dupont-Frechette, J. A., Tellock, P. P., Maher, I. D., Haisley, L. D., & Holler, K. A. (2016). Verbal memory abilities in severe childhood psychiatric disorders and the influence of attention and executive functions. *Archives of Clinical Neuropsychology, 31*(8), 934–943. https://doi.org/10.1093/arclin/acw020

Lima, A. B., Moreira, F., Gomes, M. D. M., & Maia-Filho, H. (2014). Clinical and neuropsychological assessment of executive function in a sample of children and adolescents with idiopathic epilepsy. *Arquivos de Neuro-psiquiatria, 72*, 954–959. <https://doi.org/10.1590/0004-282X20140191>

Lucas, A. J., Lewis, C., Pala, F. C., Wong, K., & Berridge, D. (2013). Social-cognitive processes in preschoolers’ selective trust: three cultures compared. *Developmental Psychology, 49*(3), 579. [https://doi.org/10.1037/a0029864](https://psycnet.apa.org/doi/10.1037/a0029864)

Lundervold, A. J., Halleland, H. B., Brevik, E. J., Haavik, J., & Sørensen, L. (2019). Verbal memory function in intellectually well-functioning adults with ADHD: relations to working memory and response inhibition. *Journal of Attention Disorders, 23*(10), 1188–1198. https://doi.org/10.1177/10870547155808

Martín Perpiñá, M. D. L. M., Viñas Poch, F., & Malo Cerrato, S. (2019). Media multitasking impact in homework, executive function and academic performance in Spanish adolescents. *Psicothema*, *31*(1), 81-87. https://doi.org/ 10.7334/psicothema2018.178.

Niermeyer, M. A., Ziemnik, R. E., Franchow, E. I., Barron, C. A., & Suchy, Y. (2019). Greater naturally occurring expressive suppression is associated with poorer executive functioning and motor-sequence learning among older adults. *Journal of Clinical and Experimental Neuropsychology, 41*(2), 118–132. <https://doi.org/10.1080/13803395.2018.1502257>

Rhodes, S. M., Booth, J. N., Campbell, L. E., Blythe, R. A., Wheate, N. J., & Delibegovic, M. (2014). Evidence for a role of executive functions in learning biology. *Infant and Child Development, 23*(1), 67–83.  <https://doi.org/10.1002/icd.1823>

Rhodes, S. M., Booth, J. N., Palmer, L. E., Blythe, R. A., Delibegovic, M., & Wheate, N. J. (2016). Executive functions predict conceptual learning of science. *British Journal of Developmental Psychology, 34*(2), 261–275. <https://doi.org/10.1111/bjdp.12129>

Rosas, R., Espinoza, V., Garolera, M., & San-Martín, P. (2017). Executive functions at the start of kindergarten: Are they good predictors of academic performance at the end of year one? A longitudinal study/Las Funciones Ejecutivas al inicio de kínder,¿ son buenas predictoras del desempeño académico al finalizar primer grado?: un estudio longitudinal. *Studies in Psychology, 38*(2), 451–472. <https://doi.org/10.1080/02109395.2017.1311458>

Semrud-Clikeman, M., Fine, J. G., & Bledsoe, J. (2014). Comparison among children with autism spectrum disorder, nonverbal learning disorder and typically developing children on measures of executive functioning. *Journal of Autism and Developmental Disorders, 44*(2), 331–342. <https://doi.org/10.1007/s10803-013-1871-2>

Taha, H. (2017). Poor executive functions among children with moderate-into-severe asthma: Evidence from WCST performance. *Frontiers in Psychology, 8*, 793. <https://doi.org/10.3389/fpsyg.2017.00793>

**The 15 Most Cited Effortful Control (EC) Articles (2013***–***2022)**

Bao, Z., Li, D., Zhang, W., & Wang, Y. (2015). School climate and delinquency among Chinese adolescents: Analyses of effortful control as a moderator and deviant peer affiliation as a mediator. *Journal of Abnormal Child Psychology, 43*(1), 81–93. <https://doi.org/10.1007/s10802-014-9903-8>

Cerda, C. A., Im, M. H., & Hughes, J. N. (2014). Learning-related skills and academic achievement in academically at-risk first graders. *Journal of Applied Developmental Psychology, 35*(5), 433–443. <https://doi.org/10.1016/j.appdev.2014.08.001>

Di Norcia, A., Pecora, G., Bombi, A. S., Baumgartner, E., & Laghi, F. (2015). Hot and cool inhibitory control in Italian toddlers: Associations with social competence and behavioral problems. *Journal of Child and Family Studies, 24*(4), 909–914. https://doi.org/10.1007/s10826-014-9901-z

Duckworth, A. L., Tsukayama, E., & Kirby, T. A. (2013). Is it really self-control? Examining the predictive power of the delay of gratification task. *Personality and Social Psychology Bulletin, 39*(7), 843–855. [https://doi.org/10.1177/0146167213482589](https://doi.org/10.1177%2F0146167213482589)

Kim, S., Nordling, J. K., Yoon, J. E., Boldt, L. J., & Kochanska, G. (2013). Effortful control in “hot” and “cool” tasks differentially predicts children’s behavior problems and academic performance. *Journal of Abnormal Child Psychology, 41*(1), 43–56. <https://doi.org/10.1007/s10802-012-9661-4>

Lin, B., Liew, J., & Perez, M. (2019). Measurement of self-regulation in early childhood: Relations between laboratory and performance-based measures of effortful control and executive functioning. *Early Childhood Research Quarterly, 47*, 1–8. <https://doi.org/10.1016/j.ecresq.2018.10.004>

Lin, W. L., Hsu, K. Y., Chen, H. C., & Chang, W. Y. (2013). Different attentional traits, different creativities. *Thinking Skills and Creativity, 9*, 96–106. <https://doi.org/10.1016/j.tsc.2012.10.002>

Lipsey, M. W., Nesbitt, K. T., Farran, D. C., Dong, N., Fuhs, M. W., & Wilson, S. J. (2017). Learning-related cognitive self-regulation measures for prekindergarten children: A comparative evaluation of the educational relevance of selected measures. *Journal of Educational Psychology, 109*(8), 1084–1102. [https://doi.org/10.1037/edu0000203](https://psycnet.apa.org/doi/10.1037/edu0000203)

Omura, K., & Kusumoto, K. (2015). Sex differences in neurophysiological responses are modulated by attentional aspects of impulse control. *Brain & Cognition, 100*, 49–59. <https://doi.org/10.1016/j.bandc.2015.09.006>

Studer-Luethi, B., Bauer, C., & Perrig, W. J. (2016). Working memory training in children: Effectiveness depends on temperament. *Memory & Cognition, 44*(2), 171–186. https://doi.org/10.3758/s13421-015-0548-9

Sulik, M. J., Eisenberg, N., Spinrad, T. L., & Silva, K. M. (2015). Associations between respiratory sinus arrhythmia (RSA) reactivity and effortful control in preschool‐age children. *Developmental Psychobiology, 57*(5), 596–606. <https://doi.org/10.1002/dev.21315>

Tiego, J., Bellgrove, M. A., Whittle, S., Pantelis, C., & Testa, R. (2020). Common mechanisms of executive attention underlie executive function and effortful control in children. *Developmental Science, 23*(3), e12918. <https://doi.org/10.1111/desc.12918>

Wang, M., Deng, X., & Du, X. (2018). Harsh parenting and academic achievement in Chinese adolescents: Potential mediating roles of effortful control and classroom engagement. *Journal of School Psychology, 67*, 16-30. <https://doi.org/10.1016/j.jsp.2017.09.002>

Zeytinoglu, S., Calkins, S. D., Swingler, M. M., & Leerkes, E. M. (2017). Pathways from maternal effortful control to child self-regulation: The role of maternal emotional support. *Journal of Family Psychology, 31*(2), 170–180. [https://doi.org/10.1037/fam0000271](https://psycnet.apa.org/doi/10.1037/fam0000271)

Zorza, J. P., Marino, J., de Lemus, S., & Mesas, A. A. (2013). Academic performance and social competence of adolescents: Predictions based on effortful control and empathy. *The Spanish Journal of Psychology, 16*. <https://doi.org/10.1017/sjp.2013.87>
